# Stable individual differences in separation calls during early development in cats and mice

**DOI:** 10.1186/1742-9994-12-S1-S12

**Published:** 2015-08-24

**Authors:** Robyn Hudson, Marylin Rangassamy, Amor Saldaña, Oxána Bánszegi, Heiko G Rödel

**Affiliations:** 1 Instituto de Investigaciones Biomédicas, Universidad Nacional Autónoma de México, Distrito Federal 04510, Mexico; 2 Laboratoire d'Ethologie Expérimentale et Comparée, Université Paris 13, Sorbonne Paris Cité, Villetaneuse 93430, France

**Keywords:** Development, Personality, Siblings, Open field test, Vocalization, Locomotion, Felis silvestris catus, Mus spicilegus

## Abstract

**Background:**

The development of ethologically meaningful test paradigms in young animals is an essential step in the study of the ontogeny of animal personality. Here we explore the possibility to integrate offspring separation (distress) calls into the study of consistent individual differences in behaviour in two species of mammals, the domestic cat (*Felis silvestris catus*) and the mound-building mouse (*Mus spicilegus*). Such vocal responses in young mammals are a potentially useful test option as they represent an important element of mother-offspring communication with strong implications for offspring survival. In addition, the neural control of vocalisation is closely associated with emotional state.

**Results:**

We found marked similarities in the pattern of individual responses of the young of both species to separation from their mother and littermates. In the domestic cat as well as in the mound-building mouse, individual differences in the frequency of calls and to a lesser extent in locomotor activity were repeatable across age, indicating the existence of personality types. Such consistencies across age were also apparent when only considering relative individual differences among litter siblings. In both species, however, individual patterns of vocalisation and locomotor activity were unrelated. This suggests that these two forms of behavioural responses to isolation represent different domains of personality, presumably based on different underlying neurophysiological mechanisms.

**Conclusions:**

Brief separation experiments in young mammals, and particularly the measurement of separation calls, provide a promising approach to study the ontogeny of personality traits. Future long-term studies are needed to investigate the association of these traits with biologically meaningful and potentially repeatable elements of behaviour during later life.

## Introduction

Interest has been growing among behavioural biologists in the existence of individual differences in behavioural phenotypes of a kind now frequently referred to as animal personality [1-5]. Once considered to be the exclusive domain of human psychologists, differences in animal personality are now considered to be the result of adaptive evolutionary processes [6-8], and to occur across a wide range of taxa [9-12]. Despite some uncertainty as to an exact definition of personality [13,14] and differences in theoretical applications of this construct, it is generally agreed that individual differences in behavioural traits need to be stable across an appreciable time span and/or different contexts to qualify [3,4,10,15,16].

More recently, attention has been drawn to the fact that there are still few studies of the ontogeny of such differences; when and how they emerge across development and how they relate to differences at later life stages [10,17-20]. This is particularly true for mammals, although an increasing number of recent studies have assessed and could successfully show the existence of personality traits in young animals around weaning by means of behavioural consistencies across time and context [21]. For example, repeated standardized tests such as open field, novel object or elevated plus maze tests have been used in young rodents before and/or shortly after weaning under laboratory conditions (laboratory rat *Rattus norvegicus *[22], guinea pigs of wild origin *Cavia aperea *[23]) or in animals captured in the field (eastern chipmunks *Tamia striatus *[24], European rabbits *Oryctolagus cuniculus *[25]). Studies have also been made in very young mammals from birth until weaning of differences in behaviour among littermates under undisturbed conditions in the litter huddle (domestic rabbits [26-28]; domestic cats *Felis silvestris catus *[29]; review in [30]). Furthermore, tests in wild animals such as flight initiation distances or responses to trapping (juvenile yellow bellied marmots *Marmota flaviventris *[31]) and handling responses of young during the nest period (European rabbits [25]) have been used to assess personality types. In a study of little brown bats (*Myotis lucifugus*), exploration in animals caught from the wild has been repeatedly tested across development using modified hole board tests [32].

Studying the development of personality in mammals is difficult for at least two reasons: the problem of testing dependent young without disturbing the often close mother-young relationship and affecting normal development; and the limited but rapidly developing and thus changing behavioural repertoire, particularly of altricial species. All this makes it difficult to identify and test biologically meaningful behavioural variables that can be followed over a longer developmental time course [10].

A notable example of behaviour largely free of such difficulties is the response of dependent young to social isolation, that is, to separation from their mother or other care givers, and in the case of polytocous species, also from littermates. Under such conditions the young of various mammals quickly start to emit persistent vocalizations (separation distress calls) of clear adaptive significance. This behaviour usually results in the arrival of the mother or other care giver(s) (e.g. humans [33]), often leading to the rapid retrieval of the young to the nest or burrow (e.g. various rodent species [34-36]; domestic cat *Felis silvestris catus *[37], own observations) or in mothers preparing to defend their young against potential predators or infanticidal conspecifics [38-41].

Vocalizations are a particularly useful behavioural measure. In mammals, distress or separation calls of dependent young typically consist of enduring trains of often high-pitched calls of variable frequency [42]. Not only can the frequency of emission and acoustic properties be readily measured and quantified [43], but the neural control of vocalizations is closely integrated with and reflects the emotional state of an individual (e.g. [44-46]; reviews in [47,48]). This is particularly the case for vocalizations associated with negative states such as alarm, fear or pain, due to the close neural connections within the brain of vocal centres, with limbic structures such as the amygdala importantly involved in the regulation of negative affect [47-49].

However, despite the clear functional significance of separation calling in young mammals and the close link to an individual's emotional state, it has scarcely been used in the study of personality in non-human mammals. One of the few published studies related to this is in cattle (*Bos taurus*), where vocalisation was quantified in repeated open field tests [45]. Furthermore, a study in lambs of domestic sheep (*Ovis aries aries*) showed that individual differences in the emission of high pitched bleats after separation were positively associated with lambs' sociability [50].

Here, we present data from two altricial mammals with rather different life histories, the domestic cat (*Felis silvestris catus*) and the mound-building mouse (*Mus spicilegus*). Domestic cats can be readily kept under semi-natural free-ranging conditions and mothers allow observation and handling of their new born young by familiar care givers without apparent protest or negative effects on the kittens' growth or survival [29,51,52]. Although altricial, the kittens are mobile from birth [53,54] and during the first postnatal month they emit persistent distress calls within seconds of being separated from their mother or nest [44,45,55]. The mound-building mouse is a monogamous altricial rodent species [56], occurring in a variety of open habitats including steppe grassland, pastures and agricultural areas of Central and South-Eastern Europe [57]. Mound-building mice can be successfully bred under laboratory conditions [58], and we have found that adolescent mice show consistent individual differences with respect to their behavioural responses in the open field and in elevated plus maze tests [59]. Both parents show retrieval behaviour when pups are displaced from the nest [60]. As it has been reported for various other rodent species [61,62], our preliminary studies have shown that mound-building mouse pups emit ultrasonic distress calls after separation from parents and littermates, well detectable at least until postnatal day 16.

It was therefore our aim in this study to investigate the existence of stable individual differences in behaviour during early development in these two species of altricial, taxonomically distant mammals. In addition, we tested whether such purported consistent individual differences were also present within litters, i.e. among siblings. We recorded individuals' responses in brief separation tests on repeated occasions before weaning using two behavioural measures, the number of separation calls and the amount of locomotor activity. In both species we expected stable individual differences in the performance of the two behaviours and a stable negative association between these; that is, we expected that more timid or fearful individuals would emit more vocalizations but show less locomotor activity ('freezing').

## Results

### Changes across age in vocalisation and locomotor activity

#### (a) Domestic cat

**Figure 1:**
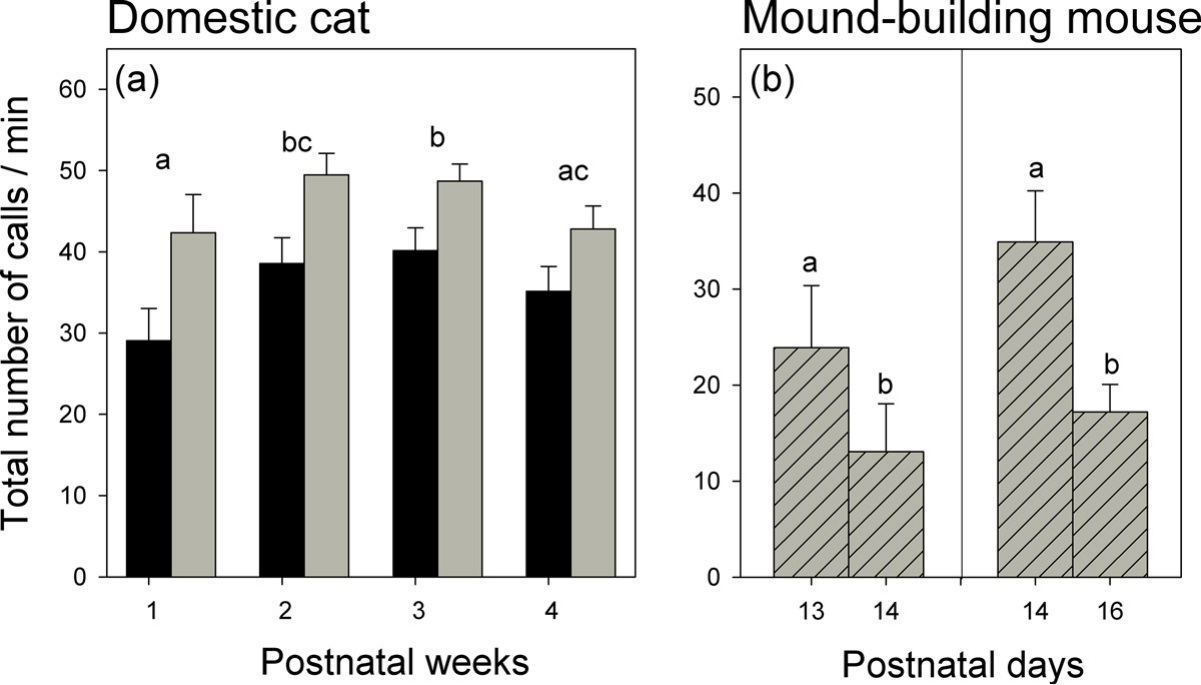
**Developmental time course of separation calls emitted by domestic kittens and mound-building mice**. (a) Kittens. Average (± SE) number of calls emitted in separation tests by males (*n *= 19; black bars) and females (*n *= 14; grey bars) during four experimental sessions across the first postnatal month. (b) Mound-building mice. Average (± SE) calls emitted in separation tests by young mice tested on postnatal days 13 and 14 (*n *= 18) and postnatal days 14 and 16 (*n *= 59). Note that data in (a) are repeated measurements, whereas 2 sets of different individuals are presented in (b). In (a), the number of calls emitted by males was significantly lower than in females. Call frequencies differed across weeks; significant post hoc comparisons are indicated by different letters. In (b), differences are significant in both subsamples; see text for statistics.

There were significant differences across age in the total number of calls emitted (GLMM for count data: $\chi_{3}^{2}=21.70$, *P *< 0.001; Figure 1a). This showed a non-linear pattern with highest values during weeks 2 and 3 (post hoc analyses in Figure 1a), and was significantly lower in males than in females ($\chi_{1}^{2}=6.29$, *P *< 0.012). The interaction between the two independent variables (age × sex) was not significant (*P *> 0.10), indicating that differences between males and females consistently occurred across all age classes. There was no association between the number of calls emitted by individual animals and their body mass.

There were no significant changes in the animals' locomotor activity during the first 4 postnatal weeks (LMM: $\chi_{3}^{2}=6.12$, *P *= 0.11). Furthermore, there was no relation between the animals' amount of locomotor activity and their body mass, and no difference on any measure of locomotor activity between males and females (all *P *> 0.10).

#### (b) Mound-building mouse

The number of vocalizations decreased significantly between postnatal days 13 and 14 as well as between postnatal days 14 and 16 (GLMM: both *P *< 0.05; Figure 1b). This decline is consistent with published results in laboratory mice (*M. musculus*), where vocalizations of pups also decreased sharply and even disappeared after about postnatal day 14 [57,58]. However, such a decrease was already apparent in our study in the independent data set between days 13 and 14, indicating that this decline might have been rather the consequence of habituation to the separation.

Locomotor activity increased significantly from day 13 to day 14 (LMM: $\chi_{1}^{2}=31.74$, *P *< 0.001) and from day 14 to day 16 ($\chi_{1}^{2}=10.95$, *P *< 0.001).

Neither the number of vocalizations nor the amount of locomotor activity were associated with pup body mass, and there were no significant differences between males and females with respect to either behaviour (all *P *> 0.10).

### Individual consistencies across age in vocalisation and locomotor activity

#### (a) Domestic cat

**Figure 2:**
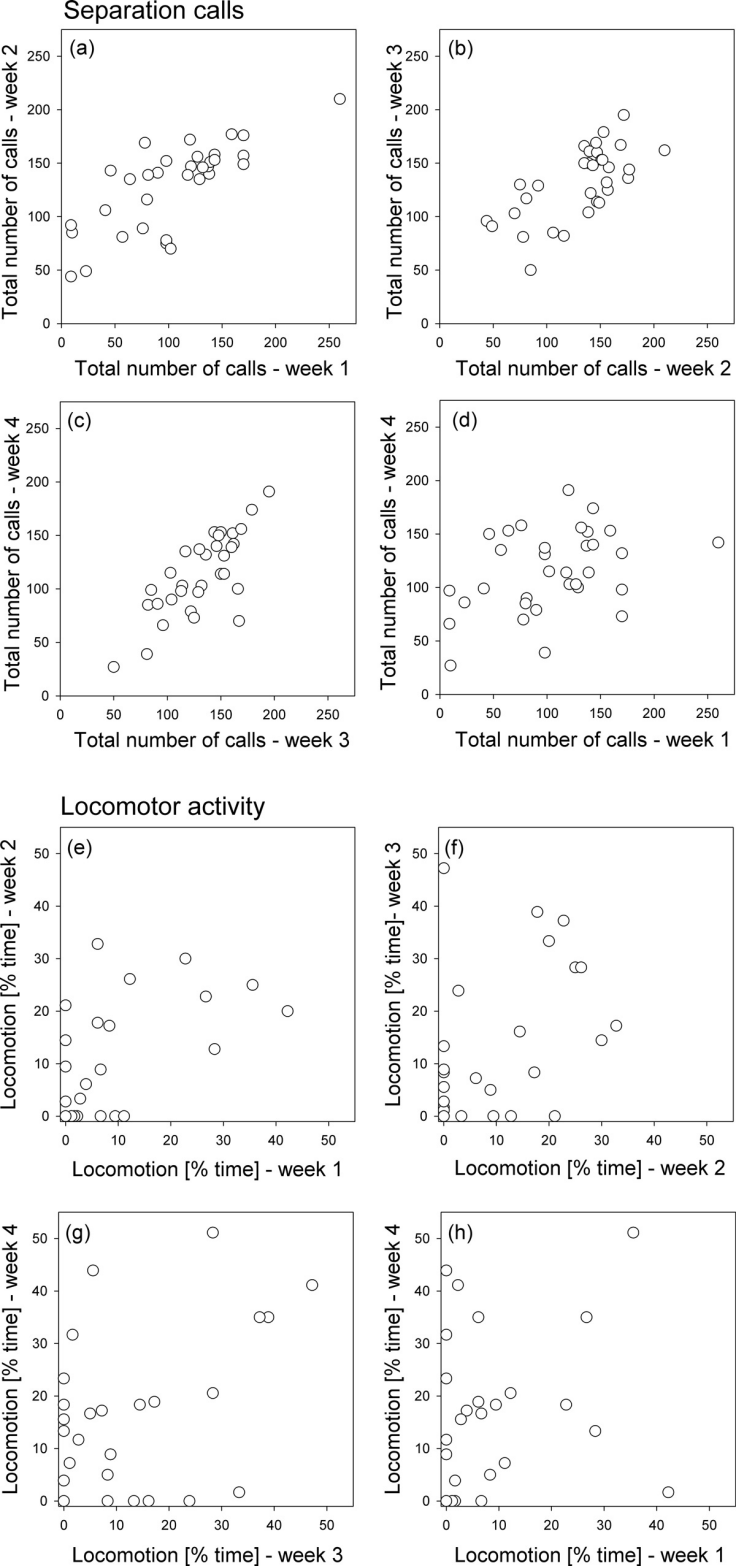
**Domestic kittens: individual differences in separation calls and locomotor activity across age**. (a-d) Separation calls. Consistent individual differences in the number of calls during four, 3-minute separation tests across the first postnatal month. (e-h) Locomotor activity. Weaker consistency in individual differences in the % time the animals spent showing locomotor activity during the four tests, with no significant correlation (h) between individual differences in the first and the final week. Each circle gives the score for an individual kitten (*n *= 33 / 8 litters). Note that the association depicted in (h) is not statistically significant; see text for details of statistics.

Individual differences in the number of calls emitted by kittens (*n *= 33) during the 3-minute separation tests were repeatable across all 4 age classes (intra-class repeatability: *R *= 0.491, *CI*_95% _= [0.299, 0.645], *P *= 0.001; Figure 2a-c), and even when only considering week 1 and week 4 (*R *= 0.298, *CI*_95% _= [0.041, 0.663], *P *= 0.015; Figure 2d). Individual differences in the time that kittens spent in locomotor activity were also repeatable across the first 4 postnatal weeks (*R *= 0.169, *CI*_95% _= [0.002, 0.349], *P *= 0.025; Figure 2e-g), although this was not the case when only testing for repeatability between weeks 1 and 4 (*R *= 0, *CI*_95% _= [0, 0.327], *P *= 0.71; Figure 2h).

We also obtained significant results when considering individual differences in behaviour with respect to litter siblings, calculated as the repeatability (among weeks 1,2,3 and 4) of the percentage deviation from the litter mean in vocalisation (*R *= 0.370, *CI*_95% _= [0.180, 0.540], *P *= 0.001) and in locomotor activity (*R *= 0.179, *CI*_95% _= [0.011, 0.347], *P *= 0.011). And again, when only taking into account data obtained during weeks 1 and 4, within-litter differences were significantly repeatable for the frequency of vocalisation (*R *= 0.298, *CI*_95% _= [0, 0.581], *P *= 0.043) but not for within-litter differences in locomotion (*R *= 0.141, *CI*_95% _= [0, 0.477], *P *= 0.209).

#### (b) Mound-building mouse

**Figure 3:**
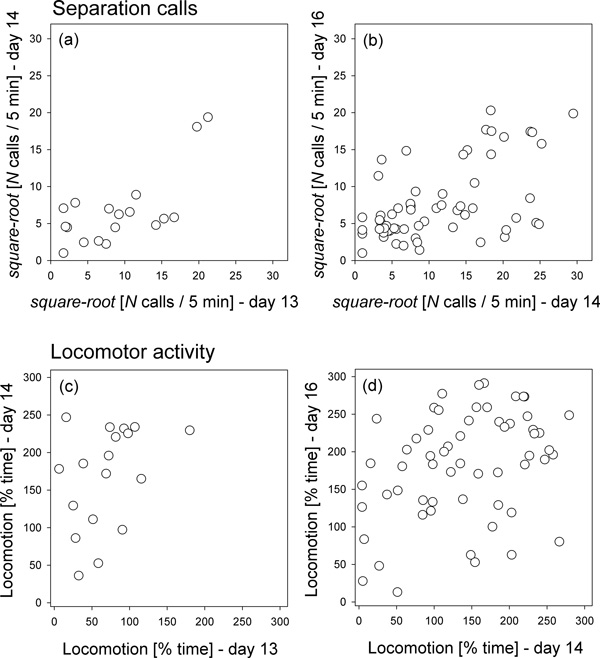
**Mound-building mice: individual differences in separation calls and locomotor activity across age**. (a,b) Consistent individual differences during two 5-minute separation tests in the number of separation calls, and (c,d) in the % time the animals spent showing locomotor activity. The frequencies of vocalization are square-root transformed for presentation. Data from repeated measures during postnatal days 13 and 14 (a,c: *n *= 18), or during days 14 and 16 (b,d: *n *= 59) are presented. Each circle gives the score for an individual mouse. Note that the association depicted in (c) is not statistically significant; see text for details of statistics.

Individual differences in the number of calls emitted by young mound-building mice during the 5-minute separation tests were repeatable between postnatal days 13 and 14 (intra-class repeatability: *R *= 0.635, *n *= 18, *CI*_95% _= [0.220, 0.877], *P *= 0.007; Figure 3a) as well as between days 14 and 16 (*R *= 0.504, *n *= 59, *CI*_95% _= [0.332, 0.745], *P *= 0.001; Figure 3b). The time that the animals spent in locomotor activity during the separation test was also repeatable between postnatal days 14 and 16 (*R *= 0.248, *CI*_95% _= [0.001, 0.488], *n *= 59, *P *= 0.036; Figure 3d), but not between postnatal days 13 and 14, when only 18 animals were tested (*R *= 0, *CI*_95% _= [0, 0.415], *n *= 18, *P *= 0.79; Figure 3c).

Also here, individuals showed repeatable differences with respect to their littermates. Within-litter intra-class repeatability with respect to the frequency of vocalisation was *R *= 0.605 (*n *= 18, *CI*_95% _= [0.212, 0.826], *P *= 0.005) between postnatal days 13 and 14, and *R *= 0.547 (*n *= 59, *CI*_95% _= [0.337, 0.701], *P *= 0.001) between days 14 and 16. With respect to locomotor activity, the within-litter repeatability was *R *= 0.421 (*n *= 18, *CI*_95% _= [0, 0.721], *P *= 0.034) between postnatal days 13 and 14, and *R *= 0.420 (*n *= 59, *CI*_95% _= [0.210, 0.631], *P *= 0.002) between days 14 and 16.

### Lack of associations between vocalisation and locomotor activity

#### (a) Domestic cat

We did not find significant associations between individual frequencies in the number of calls and the percentage time individual kittens (*n *= 33) spent in locomotor activity during testing on week 1 (GLMM for count data: $\chi_{1}^{2}=0.01$, *P *= 0.97), week 2 ($\chi_{1}^{2}=0.06$, *P *= 0.80), week 3 ($\chi_{1}^{2}=1.10$, *P *= 0.30) or week 4 ($\chi_{1}^{2}=2.78$, *P *= 0.10).

There were also no significant associations between kittens' vocalisation and locomotor activity during any of the 4 weeks when considering relative differences among littermates, i.e. the percentage deviation in these two behaviours from the litter mean (*P *> 0.10).

We tested for interactions with body mass and with sex in order to detect potentially body mass-specific or sex-specific relationships between vocalization and locomotor activity. However, all interactions were non-significant (all *P *> 0.10).

#### (b) Mound-building mouse

Also in mice we did not find significant correlations between individual differences in the number of calls emitted and the percentage of time individual pups spent in locomotor activity, either on postnatal day 13 (*n *= 18; $\chi_{1}^{2}=0.08$, *P *= 0.78), day 14 (*n *= 77; $\chi_{1}^{2}=0.01$, *P *= 0.91), or day 16 (*n *= 59; $\chi_{1}^{2}=0.28$, *P *= 0.59).

Also here, there were no indications of a significant association between the number of calls emitted and the pups' locomotor activity relative to their littermates, either during the tests on postnatal days 13, 14, or 16 (all *P *> 0.10).

We again tested for interactions with sex and with body mass, but these were non-significant in all cases (*P *> 0.10).

## Discussion

In fulfilment of one of the defining criteria for the existence of animal personality [3,4,10,63], we found stable individual differences in the behavioural responses of young kittens and mice to repeated separation from their mother, nest and littermates. In both species some individuals consistently responded by emitting a larger number of separation (distress) calls than others, and some with greater locomotor activity. Such individual consistencies in vocalisation as well as in locomotor activity were also apparent when only considering relative differences in these two behaviours among littermates. Furthermore, as revealed by our analyses, these individual differences in behaviour were not just a consequence of variation in body mass, which might be considered to potentially affect vocal or locomotor performance [22,64].

In both species, however, consistent individual differences were more evident for the emission of separation calls than for locomotor activity as shown by comparatively higher repeatabilities in calling compared to locomotion. This consistency in differences in the frequency of separation calls is in accord with similar reports in a wide range of mammals [45,50,65-67]. Thus, vocalization behaviour would seem to offer a useful means of testing for individual differences in emotionality or temperament, and particularly as in many mammals, including the cat (own observations), animals respond to separation from companions or familiar environments with distress calls across the whole lifespan ([66] for examples in other domestic animals).

Moreover, our findings seem reliable and may reflect mechanisms underlying the development of individual differences in behavioural phenotypes more generally. The two study species were housed under very different conditions, and they were tested on different schedules. Kittens were even maintained and tested under "noisy" everyday conditions, including considerable fluctuations in ambient temperature. Nevertheless, the pattern of results was highly similar for both species.

Although it is not known if the individual differences reported here are due to genetic [68,69] or experiential factors (including in utero [70,71]), their existence from such an early age in kittens suggests that there could be a genetic contribution [29]. In support of an endogenous (epi)genetic component was the difference in the number of cries emitted by male and female kittens, with males consistently emitting fewer cries across all four ages tested.

Unexpected, however, was our failure to find a correlation between individual differences on the two behaviours measured, and again in both species. Consistent individual differences across behaviours are also often considered part of the definition of personality [2,4,16]. The unexpected lack of such a relation in the present study was all the more surprising given that vocalization and locomotor activity were measured in the same context, in repeated open-field separation tests.

At one level such a disjunction might be accounted for by the problem (common in the study of behaviour) of knowing what exactly our tests, and even those as well-established as the open field, actually measure [49,72-74]. Animals may behave in seemingly similar ways for different reasons, and in seemingly different ways for the same reason. This may be particularly true for complex behaviours such as locomotion to which a wide range of neural and motivational systems contribute. Thus, some of our animals might have shown little locomotor activity from fear ("freezing") or alternatively, from a lack of motivation to move around or explore. Others might have shown high levels of activity also from fear ("panic") or alternatively, from a motivation to explore. Choosing non-arbitrary behavioural measures of evident adaptive significance might be one way to help avoid such ambiguity. Indeed, this might help explain the more robust and consistent results obtained for measures of individual differences in vocalization than in locomotor activity. Vocalizations, such as separation calls in young individuals, often have a clear functional meaning, and as mentioned in the Introduction, are the products of neural processes closely related to emotional and motivational systems [47,48].

Additionally, the lack of relation between individual differences in separation calling and locomotor activity might have been due to differential maturation of the two systems. Whereas many altricial mammals emit separation calls with facility from birth, locomotor behaviour develops more slowly and not necessarily at the same pace for all individuals, a phenomenon sometimes referred to as developmental heterochronicity [75]. This would also potentially explain the lack of a significant correlation between individual differences in locomotor activity of kittens between weeks 1 and 4, in contrast to a significant correlation in individual differences in frequency of separation calls over the same developmental time span.

A remaining issue is whether or to what extent individual differences in behaviour during early development translate into or are predictive of differences in later life [10,22,23,31,76-79]. The results of the present study, in agreement with the growing literature indicated in the Introduction, suggest that vocalizations, and particularly separation calls, might be a particularly good candidate for investigating this (see [67] for a study in human infants). A wide variety of mammals, including many domesticated and laboratory species emit separation calls during juvenile age or even across the whole life span. In addition, vocalizations provide readily quantifiable measures ranging from the simple frequency counts used here to detailed analyses of the physical properties of individuals' calls [43,80]. Furthermore, because vocalizations often have a known functional (adaptive) significance, they may better reflect an individual's behavioural, physiological and psychological state than the somewhat arbitrary test paradigms sometimes used in studies of animal personality.

## Conclusions

The separation calls emitted by many mammalian young (and in some species also by adults) when isolated from their mother, other caregivers or companions, seem to provide a particularly useful behavioural indicator for studying the ontogeny of personality. Kittens given brief separation tests once a week for the first four postnatal weeks (until the start of weaning) showed stable individual differences in the frequency of emitting such calls. This was also the case for mound-building mice although tested across a shorter developmental period. A second widely used indicator of individual differences in personality, locomotor activity, gave less consistent evidence of stable individual differences. We suggest that separation calls are particularly reliable indicators of personality because of the close neural connections between vocal and emotional systems of the brain, and because of their clear functional meaning (adaptive significance). It remains to investigate whether such differences persist into adulthood, at least in the cat which responds life-long to separation from companions or familiar surroundings with persistent isolation (distress) cries.

## Materials and methods

### Study animals and sample sizes

#### (a) Domestic cat

We collected data from 33 kittens (19 males, 14 females) of eight litters from five multiparous, crossbreed mothers kept at a private home in Mexico City [29,51,52]. The mothers had mated with local free-ranging males, which from observations were usually several different individuals for each female. Once a day they were fed commercial canned cat food and fresh meat. Water, milk, commercial dried cat food and litter trays were always available. Mothers shared the house with other intact male and female cats and were free to leave the house at will. Except when kittens were being tested (see below) mothers had free access to their young. When 8 weeks old (weaning), kittens were transferred to the cat facility at the Laboratorio de Psicobiología de Desarrollo, Instituto de Investigaciones Biomédicas, UNAM, Mexico.

#### (b) Mound-building mouse

We collected data from 77 pups (60 males and 17 females) of 23 litters, each stemming from a different parental pair (further details under *Experimental procedure *below). Studies on mound-building mice (*Mus spicilegus*) were carried out in the Laboratoire d'Ethologie Expérimentale et Comparée at the Université Paris 13 in France. The animals of the breeding stock maintained in this laboratory were descendants (16^th, ^17^th ^and 18^th ^generation) of animals caught from the wild at different sites in Hungary in 1999. We ensured the genetic variation of the breeding stock by adding some new individuals every 2-4 years, captured at the same Hungarian collection sites. Animals were kept on a 14:10 h light/dark cycle (lights off and red light on at 12:00 am) in standard polycarbonate cages (26 × 14 × 16 cm, Iffa Credo, Lyon, France), containing wood shavings as bedding. Animals had *ad libitum *access to rodent standard diet (Special Diets Services, Ext. M20, Witham, Essex, UK) and water. Temperature in the housing rooms was maintained at 21 ± 5°C, and relative humidity at approximately 50%. Except during experiments, all pups used in this study were kept with their parents and siblings in their home cage. Several cotton balls (diameter: approx. 5 cm) were always provided, which the animals used for nest building in a corner of their cage.

### Experimental procedures

#### (a) Domestic cat

Mothers gave birth in foam rubber beds 70 cm × 40 cm, lined with flannel and located in a quiet part of the house. Several hours later (and daily thereafter) we weighed the kittens on digital scales to the nearest gram. We recorded their sex, fitted each with a differently coloured neck ribbon for individual identification, and returned them to their mother until the start of testing two days later (see below). We considered the day of birth as postnatal day 1. Starting on postnatal day 3, we tested each kitten once a week with an inter-test interval of about 7 days until the end of the first postnatal month (4 test sessions per kitten, 132 sessions in total; see below). During testing, we confined the mother in a familiar transport cage in a separate room to her litter, and in random order we placed each kitten individually for 3 min in the centre of a 1 m diameter arena located in a room away from the rest of the litter. We recorded kittens' behaviour in the late morning, including vocalizations, using a digital video camera equipped with a microphone (Sony HDR-CX 100) and mounted 1.5 m above the centre of the arena. Kittens' separation calls range from about 2 to 7 kHz and are clearly audible to the human ear [45,81]. Immediately after testing, each kitten was returned to its littermates, suspended by the scruff of its neck to mimic the method of retrieval by the mother.

Throughout the study, animals were kept and treated according to the Guide for the Care and Use of Laboratory Animals of the National Institutes of Health, USA, and the National Guide for the Production, Care and Use of Laboratory Animals, Mexico (Norma Oficial Mexicana NOM-062-200-1999). The experimental procedures had no apparent effect on the general behaviour of mothers and young, kittens showed normal weight gain and all survived to the end of the study.

#### (b) Mound-building mouse

Mothers gave birth in their home cage. As we checked the nests daily for new born pups, we could determine the day of birth with an accuracy of 24 h, and considered this as postnatal day 1.

Pups repeatedly (2 times per individual, see below) underwent a 5 min separation test, where we placed them singly into an arena and recorded their vocalization and locomotor activity. Three to four pups per litter were randomly chosen and were individually marked with a permanent non-toxic hair dye (Nyanzol-D, Greenville Colorants, New Jersey, USA) on their backs in the morning (around 4 hours before the end of the white light period) of the first day of testing. Tests were carried out around 6 hours later, during the early red light period. For this, we placed each pup in a defined corner of a test arena, consisting of a rectangular plexiglas box (14.5 × 9.5 × 8.5 cm). The frequency of separation calls emitted by pre-weaned laboratory mice (i.e. in a closely related species of the same genus) usually ranges from 40 to 90 kHz [35,82]. Therefore, we recorded pups' ultrasonic calls by the use of a bat detector (Batbox Baton, Batbox LTD, Steyning, UK) fixed 5cm above the centre of the test arena. The frequency range of this detector was between 20 and 120 kHz. Recordings were automatically transformed by a division factor of 10 (i.e. 50 kHz were reduced to 5 kHz) to make them detectable for the human ear. During the experiments, recordings were saved on file for later analysis. We also recorded the pups' locomotor behaviour by a video camera (Sony HDR-XR 200), filming the arena from the side through the plexiglas as the bat detector was mounted over the arena and thus filming from above was not possible. After testing, subjects were removed from the arena, weighed to an accuracy of 0.01 g, and returned to their home cage with their parents and siblings.

As reported above, experiments were based on 77 pups born in 23 litters, each stemming from a different parental pair. A subset of pups was tested at postnatal day 13 and again at day 14 (*n *= 18 / 4 litters). Remaining subjects were tested at postnatal day 14 and at day 16 (*n *= 59 / 19 litters; 2 sessions per pup, 154 test sessions in total). We used two independent sets of animals with slightly different age classes to obtain information on the generality of our results at least across a short span of juvenile life. Our preliminary tests revealed that mound-building mouse pups show ultrasonic vocalization after separation until at least postnatal day 16. However, we did not test them before day 13 because, in contrast to the larger and well-furred kittens, isolated mouse pups cool quickly when very young, affecting their ability to vocalize [35]. Moreover, we needed to mark them individually prior to testing, which is only feasible in a minimally invasive way after they have fur at the end of the second week [57].

Animals were kept and treated according to the ethics and animal care guidelines of France (where the project was carried out) and the institutional guidelines of animal welfare. Experimental procedures were approved by the local authority for laboratory animal care and use (Comité d'Ethique en Expérimentation Animale 'Charles Darwin'; authorization codes: Ce5/2011/068; Ce5/2012/212; 00809.02). Also in the mound-building mice, the experimental procedures had no apparent effect on the general behaviour of parents and offspring. The pups showed normal weight gain and all survived to the end of the study.

### Behavioural measures

#### (a) Vocalisation

In both study species we measured the total number of calls emitted by each individual during each separation test; four 3-min sessions in kittens, two 5-min sessions in mouse pups.

#### (b) Locomotor activity

We defined locomotion as displacement of the whole body, including all four paws. In both species the occurrence of this behaviour was quantified in seconds (transformed into % observation time for statistical analysis) by analysis of video footage for each individual in each session.

### Statistical analysis

Statistical analyses were carried out using the program R, version 3.1.1 [83].

First, we tested for changes across age with respect to the number of emitted calls and locomotor activity. This was done by using linear mixed-effects models (LMM) to analyse locomotor activity (dependent variable), and generalized linear mixed-effects models (GLMM) for count (i.e. Poisson distributed) data for the analysis of the number of emitted calls (dependent variable). Vocalizations of kittens corresponded well to a normal distribution and thus we used an identity link. For data analysis of young mice, we used GLMM with a square-root link in order to adjust for the right-skewed distribution of the data. GLMMs and LMMs were calculated with the R package *lme4 *[84]. Models included multiple independent variables. In the case of kittens we tested for changes across the 4 ages (factor with 4 levels), and in the case of young mound-building mice we ran 2 different models as we had 2 different data sets, each with 2 ages (postnatal days 13 vs. 14 and 14 vs. 16; factors with 2 levels). In addition, we tested for potential differences among males and females (factor with 2 levels) and models also included individual body mass (covariate; measured directly after each test). The latter variable was considered as previous reports on pre-weaned small mammals highlight the effects of individual body mass on behavioural responses in different test situations [22,85]. Statistical models on kittens included random factors coding for litter identity and maternal identity, as several subjects were litter siblings (in total 8 different litters) or originated from the same mothers (in total 5 different mothers). In mice, we only used litter identity as a random factor (in total 23 different litters), as each litter stemmed from a different parental pair. In all cases random effects were random intercepts. In the case that differences among groups (see Figure 1a) were significant, we used GLMM with the same setting of random factors for pairwise post-hoc comparisons between the different time steps. Alpha levels were corrected for multiple comparisons by a sequential Bonferroni correction [86].

In a last section of the results, we tested for associations between individuals' frequency of vocalizations and locomotor activity (both independent variables), again using GLMM for count data. Also here we used multiple independent variables, including sex (factor with 2 levels) and body mass (covariate). Again, litter identity and maternal identity (in the case of kittens) were used as random factors. These analyses were done separately for different ages, i.e. during postnatal weeks 1, 2, 3 and 4 in kittens and on postnatal days 13, 14 and 16 in young mice. For all models we tested for interactions among the predictor variables. Non-significant interaction terms or independent variables were sequentially removed from the models before these were re-calculated. We calculated variance inflation coefficients (VIF) for all models with multiple fixed factors / covariates in order to check for (multi)colinearities among them [87]. VIF were always lower than 1.5, indicating no interfering effects of multicolinearities. We verified homogeneity of variances by plotting residuals versus fitted values for all models [88]. Whenever GLMM for Poisson distribution showed signs of overdispersion we included case-level random effects [89]. When using LMM we made sure that the model residuals were normally distributed by visually checking normal probability plots. *P*-values were extracted by Wald chi-square tests (type III).

Furthermore, we tested for repeatability of vocalization and of locomotor activity during the test sessions across age classes. We (a) analysed the total frequency of emitted calls and time that the animals showed locomotor behaviour during the experiments. In addition, we (b) calculated relative differences among littermates with respect to these 2 behaviours as the individual percentage variation from the litter mean. This was done in order to test for within-litter repeatabilities in behaviour. To this end we applied intra-class correlations calculated as the proportion of phenotypic variation that can be attributed to between-subject variation [90]. For kittens, this was done using repeated measurements of *n *= 33 individuals across 4 tests conducted during the first 4 postnatal weeks (once per week). In young mice this was done separately in two different samples: *n *= 18 individuals were tested on postnatal days 13 and 14, and another *n *= 55 individuals were tested on days 14 and 16. We used (G)LMM-based calculations of repeatability with the aid of the R package *rptR *[91]. For testing the repeatability of the frequency of vocalization we applied an intra-class correlation based on GLMM for count (Poisson distributed) data. For data on frequencies of vocalization of kittens we used GLMM with an identity link, according to the distribution of the model residuals. Repeatabilities of locomotor activities (% time, for kittens and young mice) were calculated using an intra-class correlation based on a LMM with restricted maximum likelihoods. In mound-building mice we used an intra-class correlation based on a GLMM with a square-root link in order to adjust for the right-skewed distribution of the vocalization data base obtained. Within-litter deviation in vocalisation as well as in locomotor activity was also analysed using a LMM with restricted maximum likelihoods. For all intra-class correlations we assessed 95% confidence intervals by 1000 bootstrap steps. Individual identity was used as a random factor. *P*-values were calculated by 1000 permutations.

## Declarations

Publication costs for this article were funded by the German Research Foundation (FOR 1232) and the Open Access Publication Fund of Bielefeld and Muenster University.

## Competing interests

The authors declare that they have no competing interests.

## Authors' contributions

RH and HGR initiated the study. RH, HGR and MR designed the experiments, and MR and AS performed them. HGR, RH and MR analysed the data. RH, HGR, MR and OB contributed in writing the manuscript. All authors read and approved the final manuscript.

## Funding

The study was financially supported by grants from the UNAM funding agency DGAPA (IN205513) and the Mexican national funding agency CONACYT (48692-Q) to RH, by a grant from Université Paris 13 (BQR, 2013) to HGR, and by a grant from the French-Mexican researcher exchange program ECOS-CONACYT (M14A02) to HGR and RH. MR was supported by a PhD fellowship provided by the Ecole Doctorale Galilée, Université Paris 13. AS was supported by a fellowship within the UNAM postgraduate program in Ciencias Biológicas from the Mexican funding agency CONACYT (262519), and OB was supported by the Postdoctoral Fellowship Programme (DGAPA) of the Universidad Nacional Autónoma de México.
